# Biodegradation in Composting Conditions of PBEAS Monofilaments for the Sustainable End‐Use of Fishing Nets

**DOI:** 10.1002/gch2.202300020

**Published:** 2023-04-07

**Authors:** Jungkyu Kim, Subong Park, Junsik Bang, Hyoung‐Joon Jin, Hyo Won Kwak

**Affiliations:** ^1^ Department of Agriculture Forestry and Bioresources College of Agriculture & Life Sciences Seoul National University 1 Gwanak‐ro, Gwanak‐gu Seoul 08826 Republic of Korea; ^2^ Fisheries Engineering Division National Institute of Fisheries Science Busan 46083 South Korea; ^3^ Department of Polymer Science and Engineering Inha University 100 Inha‐ro, Nam‐gu Incheon 22212 South Korea; ^4^ Research Institute of Agriculture and Life Sciences Seoul National University 1 Gwanak‐ro Gwanak‐gu Seoul 08826 South Korea

**Keywords:** biodegradable fishing net, biodegradation, composting, PBEAS

## Abstract

The development and utilization of biodegradable plastics is an effective way to overcome environmental pollution caused by the disposal of non‐degradable plastics. Recently, polybutylene succinate co‐butylene adipate co‐ethylene succinate co‐ethylene adipate, (PBEAS) a biodegradable polymer with excellent strength and elongation, was developed to replace conventional nylon‐based non‐degradable fishing nets. The biodegradable fishing gear developed in this way can greatly contribute to inhibiting ghost fishing that may occur at the fishing site. In addition, by collecting the products after use and disposing of them in composting conditions, the environmental problem such as the leakage of microplastics strongly can be prevented. In this study, the aerobic biodegradation of PBEAS fishing nets under composting conditions is evaluated and the resulting changes in physicochemical properties are analyzed. The PBEAS fishing gear exhibits a mineralization rate of 82% in a compost environment for 45 days. As a result of physicochemical analysis, PBEAS fibers show a representative decrease in molecular weight and mechanical properties under composting conditions. PBEAS fibers can be used as eco‐friendly biodegradable fishing gear that can replace existing non‐degradable nylon fibers, and in particular, fishing gear collected after use can be returned to nature through biodegradation under composting conditions.

## Graphical abstract

Under aerobic degradation conditions, nylon 6,6 fibers is non‐degradable, whereas PBEAS fishing nets show 81% degradation in 45 days. As the biodegradation proceeded, the PBEAS fiber show a clear decrease in molecular weight and mechanical properties. The carbonyl group of PBEAS fibers is discovered to be the primary site of degradation.

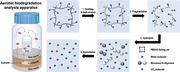

## 1 Introduction

As social interest in microplastics caused by the biodegradation of nondegradable plastics along with the depletion of petroleum resources increases, ecofriendly countermeasures are being devised.^[^ ^1^ ^]^ These measures include the replacement of petroleum resource‐based monomers with biobased monomers,^[^ ^2^, ^3^ ^]^ the development of alternative biodegradable polymer materials for reducing microplastic generation caused by nondegradable plastics,^[^ ^4^, ^5^ ^]^ and the virtuous cycle of resources through the recovery and recycling of existing plastic resources.^[^ ^6^, ^7^ ^]^ In this regard, everyday products, including many types of disposable products, are being replaced by biodegradable polymers.^[^ ^8^ ^]^ In the automobile industry, green materials that reflect eco‐friendly factors in at least one aspect from raw materials to the manufacturing process are being applied everywhere.^[^ ^9^ ^]^ In line with this trend, the paradigm is shifting to the use of biodegradable mulching films and fishing nets in the traditional agricultural and fishery industry.^[^ ^10^, ^11^ ^]^

Until now, the fishing industry has mainly used petroleum‐based hard‐to‐decompose fishing gear such as nets, pots, and traps.^[^ ^12^ ^]^ The loss of this recalcitrant fishing gear in the ocean has a serious impact on the natural environment.^[^ ^13^ ^]^ It can lead to ghost fishing,^[^ ^14^ ^]^ which causes ecosystem chaos and the deterioration of fishing ability, as well as unwanted accidents on the surface and power components of fishing vehicles. In addition, most waste gear collected after use depends on incineration, which must be disposed of after use.^[^ ^15^ ^]^ From this point of view, the development and introduction of biodegradable fishing gear material is an effective countermeasure for the sustainable development of the fishery industry and as a response to climate change in the sea environment.^[^ ^16^ ^]^ To solve the problem of unsustainable fishing circumstances, the international project called INdIGO (Innovative Fishing Gear for Oceans) is preferentially trying to develop and distribute eco‐friendly biodegradable fishing gear. In Korea, the National Institute of Fisheries Science (NIFS) has already succeeded in developing a biodegradable fishing net made of aliphatic polybutylene succinate (PBS) in 2005.^[^ ^17^ ^]^ The developed biodegradable fishing gear does not reduce fishing performance and can be biodegraded in the seawater environment to prevent “ghost fishing”. In addition, it can contribute to the virtuous cycle of carbon resources by collecting fishing gear after sufficient use at the fishing site and biodegrading it under compost conditions.

Analyzing and evaluating the biodegradation behavior of polymer materials is very important for the dissemination and circulation use of biodegradable fishing gear. This includes not only the stability of the fishing gear in storage conditions before use, marine biodegradability in actual use and loss into the ocean but also the soil biodegradability for return to nature after use. In general, in the case of biodegradable polymeric raw materials, eco‐friendliness is certified through biodegradation tests based on International Organization for Standardization (ISO) and American Society for Testing and Materials (ASTM) standards.^[^ ^18^, ^19^, ^20^, ^21^, ^22^, ^23^, ^24^ ^]^ However, to use polymer raw materials as actual products, it is essential to process them into various forms such as particles, fibers, films, and aerogels through specific polymer processing. For example, a fiber spinning process is essential for manufacturing biodegradable fishing gear, and physicochemical changes such as polymer melting, solidification, and elongation occur during this process.^[^ ^25^ ^]^

Copolymer‐based aliphatic biodegradable polymers are attractive materials that can improve various physicochemical properties of existing single‐monomer‐based polymers through changes in the composition and structure of monomers.^[^ ^26^ ^]^ From this point of view, PBEAS fiber has superior strength and elongation compared to conventional PBS fiber, which can have the advantage of preventing loss in the ocean and can be introduced into applications requiring high strength at the same time. However, studies on the decomposition behavior of these copolymers under composting conditions are still lacking. In addition, most of them present only basic biodegradation evaluation results based on mass reduction;^[^ ^27^ ^]^ thus, the current understanding of mechanisms through physicochemical characteristics analysis of polymer materials according to biodegradation is insufficient.

Herein, we experimentally proceeded by selecting biodegradation in compost conditions as the disposal process for PBEAS monofilaments after use. The biodegradation rate was evaluated based on the amount of carbon dioxide (CO_2_) emission using a laboratory‐scale aerobic biodegradation analyzer.^[^ ^28^ ^]^ The obtained biodegradation degree results were compared with actual weight loss to confirm the reliability of the evaluation method. Changes in the physicochemical properties of PBEAS fibers before and after biodegradation were investigated through molecular weight, morphology, and mechanical and thermal characteristic analysis to elucidate the biodegradation mechanism.

## 2 Results and Discussion

**Figure 1:**
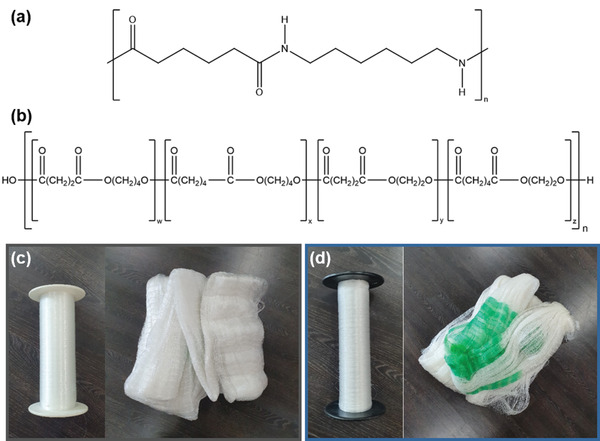
Conventional nylon 6,6‐based fibers and developed biodegradable PBEAS fibers for fishing nets. a–c) Image and chemical formula of nylon 6,6 fibers and b,d) PBEAS fibers, respectively.

Polyamide 6.6, which is commonly named as nylon 6,6, has a half‐bonded amide (‐CONH‐) bond in the backbone and is synthesized by the polycondensation of hexamethylene diamine and adipic acid.^[^ ^29^ ^]^ It is known that these polyamide structures are rarely biodegradable, despite having a structure similar to that of natural proteins and synthetic polypeptides.^[^ ^30^ ^]^ The strong durability of polyamide against such a biodegradation environment is caused by the crystalline nature of the polymer chain‐originated symmetrical molecular structure and the strong intermolecular cohesive force due to hydrogen bonding. Meanwhile, in the case of PBEAS, the polymer chain is composed of repeating units of butylene succinate, butylene adipate, ethylene succinate, and ethylene adipate, and it is known that the strength and elongation are improved compared to the conventional PBS homopolymer.^[^ ^11^ ^]^ Above all, regardless of its copolymeric chemical composition, the PBEAS copolymer has biodegradable aliphatic ester groups, so it can be used as an eco‐friendly alternative to existing nondegradable plastic materials. **Figure** **1** shows the chemical formulas of conventional nondegradable nylon 6,6 and biodegradable PBEAS, monofilaments, and manufactured fishing nets via the weaving process. Compared with the existing nylon 6,6 fiber, there is no significant difference between the monofilament and the actual manufactured fishing gear and color; therefore, it is judged that the sense of heterogeneity can be reduced even when applied to the actual fishing environment.

**Figure 2:**
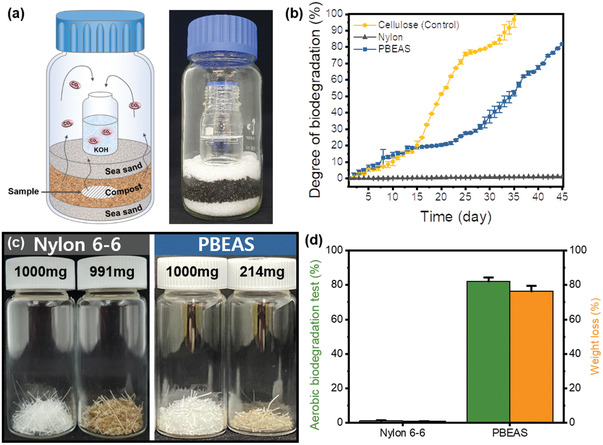
a) Schematic of laboratory‐scaled CO_2_ emission analyzer and its images, b) biodegradation experiment results, c) a residual sample image, and d) a graph comparing the degradation rate through mass loss measurement.

**Figure** **2**a shows a laboratory‐scale aerobic biodegradation analyzer manufactured based on ISO 14855‐2. In this biodegradation experiment, composting conditions were used, which are known to show rapid biodegradation behavior by increasing the amount of microbial strains compared to the existing soil conditions through the activation process. The generated carbon dioxide collects KOH present in the upper part of the compost, and the biodegradation rate according to the incubation period can be indirectly calculated through titration or quantification through additional chemical reactions. Figure 2b shows the results of biodegradation experiments of nylon 6,6 and PBEAS fibers through CO_2_ emission tester for laboratory scale. Cellulose was added as a control to verify the effectiveness and significance of the experiment. In the case of cellulose, biodegradation progressed gradually as the degradation time increased, and nearly 100% biodegradation occurred at 34 days. This means that the biodegradation experiment by CO_2_ capture can verify the actual biodegradation efficiency. In addition, the reason why the result of more than 100% after 34 days is because the distribution of microbial strains differs from that of the control group as biodegradation of cellulose occurs under composting conditions. In the case of nylon 6,6 fibers, no significant additional CO_2_ generation was observed compared to the blank environment to which the sample was not included. This means that nylon 6,6 fibers cannot undergo biodegradation by microorganisms under composting conditions, which is consistent with previous results. Alternately, in the case of PBEAS fiber, gradual decomposition occurred up to the initial 15 days, and additional continuous decomposition occurred after the 25th day, showing a total decomposition degree of close to 81% during the decomposition period of 45 days. This two‐step biodegradation pattern is due to the following reasons. First, hydrolysis of PBEAS and degradation of soluble substances occur preferentially at the beginning of biodegradation. Thereafter, as the mineralization by microorganisms is promoted by the increase in oligomeric or low molecular weight substances, the entire biodegradation rate could be accelerated. Measuring weight loss is the most intuitive way to assess biodegradability. Figure 2c shows the shape and weight loss results of actual nylon 6,6 and PBEAS fibers before and after the degradation process. In the case of nylon 6,6 fibers, although color change was observed after being buried in the composting environment, the mass change did not occur significantly, whereas PBEAS showed a large mass decreased from 1000 to 214 mg. To confirm the accuracy of the biodegradability measurement result through the laboratory‐scaled CO_2_ emission test, Figure 2d shows the biodegradation degree result based on the CO_2_ evolution and weight reduction. As shown in the figure, in the case of the PBEAS fiber, the weight reduction and CO_2_ evolution results showed a low difference in biodegradability within 5%, which proves that CO_2_ capture by the aerobic biodegradability evaluation experiment is an effective tool to prove the actual biodegradability. In the case of weight loss‐based degradation experiments, a sample must be taken, and its weight must be measured every period, which has a disadvantage in that it is difficult to accurately measure the weight due to contamination or elements attached to the sample species.^[^ ^31^ ^]^ In addition, if the sample deteriorates or collapses and loses structural stability, it is difficult to recover the sample. Alternately, the CO_2_ emission measurement experiment has the advantage of being able to measure the degree of biodegradation continuously without sample recovery.^[^ ^32^ ^]^ The latter part discusses the changes in physicochemical properties according to the biodegradation behavior of PEBAS fibers by dividing them into molecular weight, crystal properties, mechanical properties, and thermal properties.

**Figure 3:**
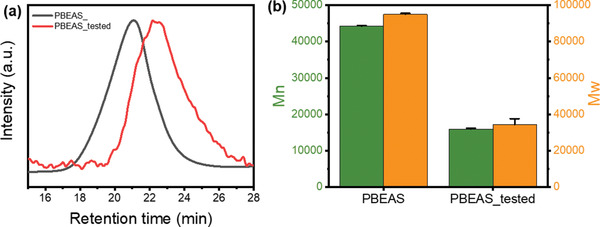
a) SEC chromatogram and b) molecular weight change of PBEAS before and after biodegradation. Mn and Mw denote number and weight average molecular weight, respectively.

Tracking the molecular weight change of polymers due to degradation provides useful information for elucidating the biodegradation mechanism.^[^ ^33^, ^34^ ^]^ It is possible to distinguish bulk or surface erosion by checking the molecular weight reduction pattern according to the biodegradation phenomenon.^[^ ^35^ ^]^ **Figure** **3**a shows the SEC chromatogram of PBEAS fiber before and after decomposition in a composting environment and the result of the molecular weight change obtained through this process. The SEC results showed that a significant decrease occurred in the molecular weight of the PBEAS fibers over degradation time. Originally, PBEAS fibers showed unimodal peaks in SEC, but in the case of fibers that have undergone biodegradation in a compost environment, the molecular weight dispersity increases, and oligomer formation can be confirmed due to polymer chain scission.^[^ ^36^, ^37^ ^]^ A significant decrease in molecular weight indicated bulk degradation. Bagheri et al. observed molecular weight changes according to the biodegradation behavior of poly(3‐hydroxybutyrate) (PHB).^[^ ^38^ ^]^ When the degradation rate was less than 10%, no molecular weight change was observed, which was presumed to be the result of surface erosion. Alternately, when the degradation degree was 25% or more, the bulk degradation phenomenon was verified through the multimodal shift of the chromatogram and the decrease in molecular weight.

**Figure 4:**
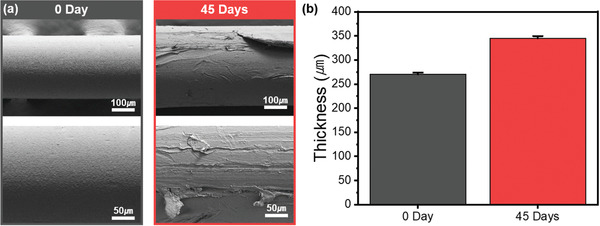
a) SEM image and b) thickness change before and after biodegradation of PBEAS fibers.

Changes in the morphology of the PBEAS fiber surface after biodegradation experiments were observed through FE‐SEM images (Figure 4). The pristine PBEAS fiber had a circular shape, and the average diameter was ≈274 µm. Additionally, the surface was smooth and uniform. Alternately, in the case of fibers that underwent biodegradation under composting conditions for 45 days, the diameter increased to 348 µm because the soil conditions were alkaline. It is generally known that alkaline conditions cause swelling and subsequent hydrolysis of polybutylene succinate (PBS) and polybutylene adipate‐co‐terephthalate (PBAT) fibers.^[^ ^39^ ^]^ It is considered that this swelling phenomenon in alkaline conditions occurred in the PBEAS copolymer. The most dramatic change is the change in surface morphology. As shown in the **Figure** **4**a, in the case of PBEAS fibers that have undergone biodegradation under composting conditions, the surface has changed to roughness, and cracks and even breakage of the fiber surface were observed. The increase in the surface area according to the change in the roughness of the surface will facilitate contact with the microbial community under composting conditions, thereby promoting biodegradation.^[^ ^40^, ^41^ ^]^

**Figure 5:**
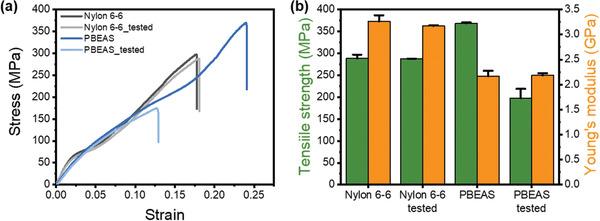
Comparison of mechanical properties of biodegraded nylon 6,6 and PBEAS: a) tensile stress–strain curves, b) tensile strength and Young's modulus. The error bars represent the s.d. of the different test (*N* = 5).

Changes in the mechanical properties of polymer materials are greatly affected by biodegradation as well as physical erosion behavior.^[^ ^42^ ^]^ In general, because there is no change in the energy dissipation behavior of nondegradable polymers unless erosion by the external environment occurs, a decrease in mechanical properties does not occur. However, in the case of biodegradable polymers, as they are exposed to a degradation environment, a decrease in molecular weight occurs. In addition, when erosion occurs in the degradation environment, an inevitable decrease in mechanical properties appears. To confirm the change in the mechanical properties of the biodegradable fiber according to the compostable degradation behavior, the tensile properties before and after degradation were measured using UTM, and the results are shown in **Figure** **5**. Because a sample fiber of a certain length or longer is required to measure the tensile strength of the fiber, when performing the aerobic biodegradation analysis experiment, a 15 cm fiber was additionally added and biodegraded to proceed with the tensile test. The initial nylon 6,6 fibers exhibited a tensile strength of 280 MPa and a modulus of 3.4 GPa. These tensile properties of nylon 6,6 fibers are similar to the previously reported nylon fibers for gillnets.^[^ ^43^ ^]^ As expected, no significant changes in tensile properties were observed before and after biodegradation of nylon 6,6 fibers. This is because, as mentioned above, nylon 6,6 fibers did not show obvious biodegradation behavior, such as mass loss, under composting conditions. On the other hand, the biodegradable PBEAS fiber showed a tensile strength of 358.4 MPa and a Young's modulus of 2.3 GPa, indicating that PEBAS fiber can sufficiently replace the existing nylon 6,6 fiber in the actual fishing environment. Moreover, in the case of PBEAS fibers subjected to biodegradation under composting conditions, the tensile strength showed a significant decrease of 58%, which is a result of the decrease in molecular weight and surface erosion. In addition, the change in Young's modulus before and after biodegradation of PBEAS fibers was insignificant. This is considered to be because the biodegradation of PBEAS fibers does not significantly affect the crystallinity and crystal structure of bulk fiber.

**Figure 6:**
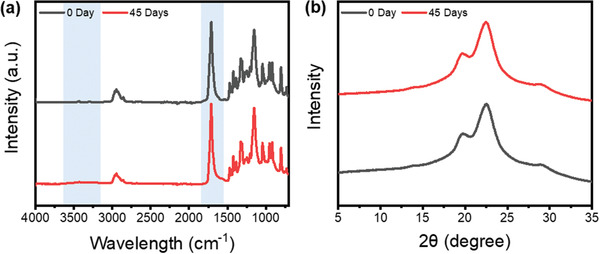
Effect of compost burial duration up to 45 days on the chemical functional groups and crystallinity of PBEAS: a) FTIR and b) XRD spectra.

To examine the change in chemical functional groups according to the degradation behavior of PBEAS fibers under compost conditions and the results are shown in **Figure** **6**, FTIR spectra before and after degradation were measured, and the results are shown in Figure 6 (a). Representative aliphatic biodegradable polymers, including PBEAS, PBS, PES, and PBAT, show complex and strong peaks at 1700–1800 cm^−1^, which is a mixture of three different carbonyl (C=O) stretching modes at different peak positions.^[^ ^44^ ^]^ For example, in the case of PBS, the band at 1736 cm^−1^ corresponds to the stretching mode of C=O in the soft‐amorphous part, the band at 1720 cm^−1^ is assigned to stretching in the hard‐amorphous part, and the band at 1714 cm^−1^ corresponds to the crystalline part.^[^ ^45^ ^]^ As shown in the figure, in the case of the PBEAS fiber, it can be expected that PBEAS has a crystalline structure through the strong peak at 1710 cm^−1^. This crystalline structure is due to molecular ordering in the fiber direction in the drawing after the melt spinning process. Meanwhile, a peak corresponding to the characteristic peak C—O stretching vibrations at 1150 cm^−1^ also appeared. As biodegradation progressed, there was no change in the position of the carbonyl peak, but a decrease in signal strength occurred. This means that biodegradation under compost conditions does not change the crystalline nature of PBEAS fibers, but the carbonyl group is the main degradation site.^[^ ^46^, ^47^ ^]^

The XRD pattern of PBEAS fibers in the 2*θ* range of 10°–40° is shown in Figure 6b. The XRD pattern of the pristine PBEAS fiber showed strong diffraction peaks at 2*θ* at 22.5° and weak peaks at 2*θ* at 19.5°, and 29.4°, indicating the formation of monoclinic crystals and diffraction from the three planes (0 2 0), (1 1 0), and (1 1 1), respectively.^[^ ^48^ ^]^ Even in the case of PBEAS fibers subjected to biodegradation for 45 days, peak positions and intensities similar to those of neat PBEAS were observed. This means that the biodegradation behavior of PBEAS under composting conditions does not cause a change in the crystal structure, and the difference in the biodegradation rate between the bulk and amorphous regions is also not large. This result can also help to interpret the mentioned mechanical strength results. Despite biodegradation, the change in the crystal structure and crystallinity of the polymer was insignificant, so there was no change in the initial modulus value of the tensile test. However, the surface morphology and the occurrence of cracks due to the swelling and decomposition of the fibers generated in the compost condition could have a significant effect on the reduction of the overall tensile strength of the fibers.^[^ ^49^ ^]^

**Figure 7:**
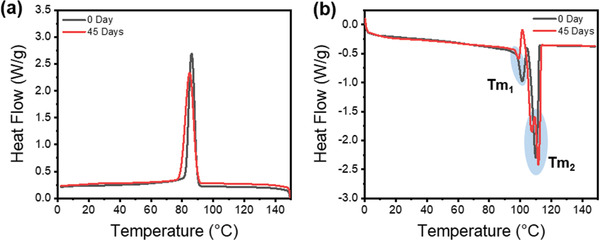
DSC cooling and heating curves of PBEAS in compost burial test for 45 days.

Through cooling and heating experiments using DSC, changes in thermal properties before and after decomposition of PBEAS fibers were investigated (**Figure** **7**). For the flat PBEAS fibers, a single crystallization peak appears during the cooling process, whether degradation occurs or not.^[^ ^50^ ^]^ In the case of PBEAS fibers that have undergone biodegradation, the *T*_c_ tends to decrease slightly, indicating slow crystallization. This decrease in *T*_c_ is thought to be because the rate of diffusion of polymer chains into crystal nuclei is hindered by residual impurities present after biodegradation. Meanwhile, the melting behavior of the polymer material can be confirmed from the DSC heating scan result.^[^ ^51^ ^]^ The PBEAS fiber shows a double melting peak, which is thought to be due to melting recrystallization by heating due to incomplete crystallization during the cooling process. Meanwhile, as biodegradation progresses, the intensity of the lower temperature Tm_1_ decreases because the incomplete crystalline structure might act as a biodegradation site. The calculated crystallinity was 40.6% before degradation to 41.7% after degradation. This indicates that the biodegradation of PBEAS fibers occurs in bulk rather than preferentially and selectively in crystalline and amorphous regions, which is consistent with the XRD results.

**Figure 8:**
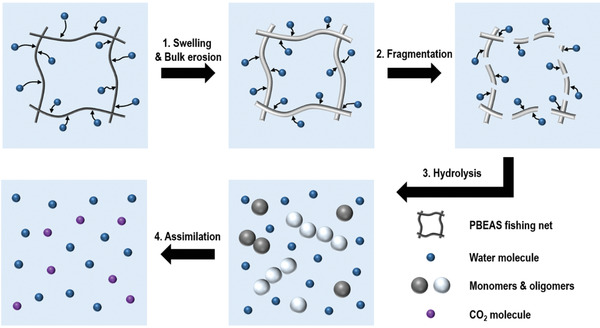
Schematic representation of the different steps in PBEAS biodegradation.

The biodegradation of PBEAS fishing nets proceeds in gradual steps under compost conditions. First of all, water molecules penetrated into the polymer structure, causing the fibers to swell and break hydrolytically unstable bonds of the amorphous phase such as ester bonds. The molecular weight decrease of the polymer occurred due to bulk erosion, and the change in molecular weight was analyzed through SEC measurement. After bulk erosion of PBEAS, biotic or abiotic hydrolysis occurs on the fragment to reduce molecular weight and form oligomers, dimers, and monomers. Finally, the decomposition products were used as carbon and energy sources by enzymes, producing CO_2_ and water as end products. CO_2_ generated by bioassimilation was measured through an aerobic biodegradation test. The described biodegradation mechanism of PBEAS fishing net is shown in **Figure** **8**.

## 3 Conclusion

In this study, the biodegradation behavior of PBEAS fiber developed as an eco‐friendly fishing net material was confirmed under composting conditions, thereby reflecting how such material is discarded into the environment after use. As a result of a biodegradation test according to ISO 14855‐2, an international standard of a laboratory scale, 82% of biodegradation occurred in PBEAS fibers for 45 days based on CO_2_ emissions, which was consistent with the weight loss result (78.4%). With the progress of biodegradation, PBEAS fibers showed a rapid molecular weight reduction of 70–80%. In addition, PBEAS fibers showed swelling under compost conditions, and cracks due to the combination of hydrolysis and biodegradation were also observed. As a result, a sharp decrease in tensile strength was observed after the biodegradation experiment. As a result of tracking the functional group change before and after degradation through FTIR, the carbonyl group of PBEAS fibers was found to be the main degradation site. As a result of confirming the crystal structure change of PBEAS fibers through XRD and DSC analysis, there was no significant change in crystallinity and crystal properties even after decomposition. This means that simultaneous biodegradation occurs in the crystalline and amorphous regions. Alternately, and most importantly, it was strongly confirmed that the existing nylon 6,6 fiber, which is a material for fishing gear, did not decompose through CO_2_ emission, weight loss, and deterioration of mechanical strength. The results suggest that biodegradable PBEAS fiber can be an effective alternative to the existing polyamide‐based fishing gear material and contribute to a virtuous cycle of resource biodegradation after use.

## 4 Experimental Section

### Materials

PBEAS and nylon 6,6 (as a negative control) fishing nets and monofilaments were kindly provided by National Institute of Fisheries Science (Busan, Korea). The molar ratio of succinic acid and adipic acid, which are dicarboxylic acid components, was 97:3, and the molar ratio of 1,4‐butanediol and ethylene glycol, which are glycol components, was 98:2. The reaction input molar ratio of the dicarboxylic acid component and the glycol component was 1: 1.25. The mean diameter of each fishing net is 280 µm. Cellulose powder suitable for thin‐layer chromatography (TLC) grade with a particle size of less than 20 µm was obtained from Sigma–Aldrich. Fertilizer and rabbit feed for preparing compost were provided from KG Chemical and Hagen, respectively. Sea sand, potassium hydroxide (KOH), and barium chloride (BaCl_2_) for measuring the biodegradability of the fishing net were supplied from Daejung (Siheung, Korea).

### Preparation of Controlled Compost for the Biodegradation Test

Controlled compost for biodegradability evaluation was produced based on ISO 14855‐2.^[^ ^52^ ^]^ The fertilizer (Stevia Gold, KG chemical ltd. Korea) was sieved using a 20‐mesh sieve and placed at room temperature to prevent drying. Rabbit feed was introduced in the fertilizer as a nutrient source for microorganisms, and sea sand was put inside the compost to create a homogeneous condition and better aerobic environment. The controlled compost was manufactured with water content of over 80% by adding an appropriate amount of water to this mixture, where the water content was determined by the weight ratio of water to solid compost. The mixed compost was activated for 2 weeks to obtain mature compost. The compost in the activation stage was mixed once a day, and the water content was adjusted to 65 RH% at 35 °C. In this step, the activation behavior of the compost was confirmed by tracking the temperature change, and the compost that had undergone the thermophilic phase at a temperature of 58 °C or higher was collected and used in the actual experiment.

### Biodegradation Test

A laboratory‐scale aerobic biodegradation test apparatus was prepared to analyze the degradation of PBEAS gear under compost conditions. The biodegradation behavior in composting conditions of the PBEAS fishing net was analyzed using a laboratory‐scale aerobic biodegradation test apparatus. Activated compost(15 g) and 1 g test materials chopped to less than 1 cm were added to a large 1000‐mL flask, and 15 g of sea sand wetted with water was installed above and below the compost to maintain the moisture content of the compost. Finally, a vial containing 50 mL 0.5 n KOH solution was positioned on the upper layer to capture CO_2_ generated as a decomposition product of the PBEAS. The biodegradation test of PBEAS was carried out by placing the flasks in an incubator set at a temperature of 58 ± 2 °C and humidity of 50–55 RH% without diffuse light. TLC‐grade cellulose powder was applied as a positive control, and a nylon 6,6 fishing net cut to the same size as a PBEAS fishing net was put as a negative control to evaluate the degree of degradation. Biodegradability analysis for all samples was repeated three times.

The biodegradation test period was set to 45 days, and the biodegradability of the PBEAS was calculated by quantifying the mass of CO_2_ generated by the degradation of the sample per day. Quantification of the emitted CO_2_ during the biodegradation of the sample was achieved through trapping with 0.5 n KOH solution. This reaction is shown below:

(1) $$2\mathrm{KOH}+{\mathrm{CO}}_{2}\to K_{2}CO_{3}+H_{2}O$$

The vial containing the CO_2_‐captured KOH solution was removed from the flask, and 1 n BaCl_2_ solution was poured to obtain BaCO_3_ salt. This reaction is displayed below:

(2) $$K_{2}CO_{3}+BaCl_{2}\to BaCO_{3}+2KCl$$

The BaCO_3_ salt produced from CO_2_ generated as a result of biodegradation of the sample appeared in the form of a turbid white precipitate, and after centrifugation, the dry weight was measured to calculate the amount of CO_2_ emission of the sample. The biodegradation rate of a sample in each flask was determined as the percentage of the material carbon content converted into CO_2_ according to the following equation (Equation 3):

(3) $$\mathrm{Biodegradation}\;\left(\%\right)=\left({\left(CO_{2}\right)}_{T}-{\left(CO_{2}\right)}_{B}\right)/\mathrm{ThC}O_{2}\times 100$$

where (CO_2_)_T_ is the amount of CO_2_ evolved in the tested sample and (CO_2_)_B_ is the amount of CO_2_ evolved in the blank flask. ThCO_2_ is the theoretical quantity of CO_2_ emitted in each flask, which can be estimated using the formula below:

(4) $$ThCO_{2}=M_{Total}\times C_{Total}\times 44.01/12.0107$$

where M_Total_ is the total dry mass of test material introduced into the compost, *C*_Total_ is the relative carbon content of the test material, determined from the chemical formula or from elemental analysis, expressed as a mass fraction, and 44.01 and 12.0107 are the molecular and atomic mass of CO_2_ and carbon, respectively. The calculated ThCO_2_ values of cellulose and nylon 6,6 as control groups were 1.629 g and 2.337 g, respectively, and the ThCO_2_ value of PBEAS, the test material, was 2.047 g.

To obtain a biodegradation rate tested for 45 days, the emitted CO_2_ was plotted versus the biodegradation time as a percentage of the converted material carbon content. In addition, after the biodegradation experiment, the remaining sample was taken and the mass was measured to obtain a weight loss result.

### Analytical Characterization

*Molecular Weight Analysis*: The molecular weight distribution of the PBEAS fishing net biodegraded in the activated compost condition was evaluated using Size exclusion chromatography (SEC, NEXERA, Shimadzu, Japan). PBEAS was dissolved in HPLC grade chloroform, filtered using a 0.45‐µm PTFE syringe filter and evaluated at a flow rate of 1 mL min^−1^. Polystyrene standards with a molecular weight range of 266–65000 Da were utilized for calibration.

*Morphological Properties*: The surface characteristics of PBEAS fishing nets before and after biodegradation were observed using a field emission‐scanning electron microscope (FE‐SEM, SUPRA 55VP, Carl Zeiss, Germany) at 5‐kV accelerating voltage and SE2 mode. Samples were coated with a fine platinum layer (10 nm) by ion sputtering before SEM analysis. The diameter changes of PBEAS fibers before and after biodegradation were measured using ImageJ software. For fiber diameter, the mean and standard deviation were obtained from three FE‐SEM images.

*Mechanical Properties*: To measure the mechanical properties after decomposition of the fishing net, PBEAS filaments were subjected under the same conditions as the aerobic biodegradation device for 45 days. The tensile properties of PBEAS were evaluated using a universal testing machine (UTM, SC34‐1, Instron, USA). Before tensile properties measurement, All samples were oven‐dried in a 60 °C for 24 h. Each specimen was trimmed to a length of 15 cm, and the thickness of the samples was measured using a thickness tester (CHY‐C2A, Labthink, China). In the test setup, the initial clamping distance was set to 50 mm, and the cross‐head speed was 50 mm min^−1^. Five specimens for each sample were tested, and the average value and standard deviation of each sample were determined.

*Fourier Transform Infrared Spectroscopy*: The chemical functional group alterations of PBEAS samples before and after biodegradation under compost conditions were examined using FTIR analysis. The spectrum of each sample was collected using ATR‐FTIR (Nicolet summit, Thermofisher scientific, USA). Each sample was mounted directly on the crystal surface and scanned 64 times with a resolution of 8 cm^−1^ in the range of 4000–700 cm^−1^.

*X‐Ray Diffraction (XRD)*: An X‐ray diffractometer (D8 Advance, Bruker, Germany) was used to analyze the crystallinity change of the PBEAS fishing net before and after decomposition. The XRD patterns were recorded in reflection mode in Cu‐K*α* radiation (*λ* = 1.5418 Å). XRD measurements were performed over the 2*θ* range from 10° to 40°, and the pressure and current of the generator were 10 kV and 1000 µA, respectively.

*Differential Scanning Calorimetry (DSC)*: DSC thermograms of the PBEAS fishing net before and after biodegradation were recorded on a Discovery DSC calorimeter (TA Instruments, USA). The specimens were placed in an aluminum pan under a nitrogen‐purged atmosphere and heated to 150 °C at a flow rate of 10 mL min^−1^ for 5 min.^[^ ^53^, ^54^ ^]^ The sample in which the thermal history was erased was cooled at a rate of 10 °C min^−1^ to 0 °C and heated at 10 °C min^−1^ to 150 °C. The crystallinity of PBEAS before and after decomposition can be compared by using the crystallization peak of the DSC curve. The crystallization temperature (*T*_c_) and melting temperature (*T*_m_) were derived from the cooling scan and second heating scan, respectively. Crystallinity (X_c_) of PBEAS was calculated by the second heating ramp from DSC, which can be determined using the formula below:

(5) $$X_{c}=\Delta H_{m}/\Delta H_{m}^{0}\times 100\%$$

where *ΔH*_m_^0^ was the theoretical value of 100% crystallized PBEAS as 110.5 Jg^−1^.

## Conflict of Interest

The authors declare no conflict of interest.

## Data Availability

The data that support the findings of this study are available from the corresponding author upon reasonable request.
